# Supplementary material for: Evaluation of a digital entomological surveillance planning tool for malaria vector control: Three country mixed methods pilot study
Source: PLoS One. 2025 Mar 10;20(3):e0303915. doi: 10.1371/journal.pone.0303915 (PMC11892875; doi:10.1371/journal.pone.0303915)
Supplement: S1 Text — S1_eSPT Explainer & Download. Further information about the eSPT software and directions on how to access it. (DOCX) [file pone.0303915.s001.docx]

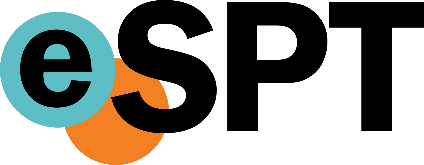
Digital Entomological Surveillance Planning Tool

The ESPT is a decision-support tool for planning entomological surveillance activities, interpreting entomological data, and guiding programmatic vector control decisions. The ESPT prioritizes entomological surveillance indicators and activities across transmission settings, geographic areas (sentinel sites versus transmission foci), and levels of program capacity. The ESPT considers how these indicators and activities influence national malaria program decisions about entomological surveillance planning and vector control response.

The ESPT includes practical approaches and priority indicators (i.e., minimum essential indicators) to help answer program questions about local transmission drivers, gaps in protection with current vector control interventions (e.g. insecticide resistance, outdoor biting, etc.), and selecting supplemental vector control interventions to address gaps in protection. In turn, these data, in combination with epidemiological and other data, will help programs tailor vector control solutions, reduce vector populations and human-vector contact, and drive down transmission. The ESPT also includes indicators and methods to improve understanding of human behavior as it relates to increased exposure to infectious mosquito bites, high risk populations that may be contributing to transmission but not accessing malaria preventative and treatment services, and relative costs of entomological surveillance activities since most countries are working with limited resource envelopes.

# Introducing the eSPT

The eSPT walks the user through a step-by-step process to develop an entomological surveillance plan, with guidance on question formulation, method selection and sampling design. During this process, users are encouraged to develop their own approach to surveillance, which they input into the software as text or by selecting pre-defined methods. The resulting plan can be exported as a formatted Word document. Further guidance is provided in the eSPT through a series of interactive decision trees. The eSPT is available for free download from the [**Malaria Elimination Initiative Website**](https://shrinkingthemalariamap.org/tool/entomological-surveillance-planning-tool-espt) and is compatible with MacOS and Windows operating systems. The eSPT is currently available in English, French and Portuguese.

# Developed by Experts

The eSPT was developed by a team of subject experts and a professional game development company.

Professor Neil Lobo – Neil’s research focuses on mosquito-borne disease transmission, with studies both in the lab as well as in the field. He has worked on research related to the entomology and epidemiology of diseases like malaria and Dengue, including transgenics, genomics, vector species compositions, vector bionomics, control strategies, intervention evaluation, vector population biology, and, human behavior and epidemiology spanning laboratory to field conditions.

Dr Michael Coleman - Michael’s research focuses on methods to improve the surveillance of vectors of human disease, and effective use of data to make informed decision on disease control and elimination. This has included establishing large scale surveillance programmes in Africa and India.

Dr Charlotte Hemingway – Charlotte’s research focuses on the use of digital technology and game design for health system benefit and health related behaviour change. Since 2014, she has worked closely with international organisations and professional game developers to co-produce and evaluate game-based interventions for Malaria control in sub-Saharan Africa and HIV prevention in Southeast Asia. Through this work she’s established and published guiding principles for the development of digital tools in the context of global health.

Dr Steven Gowelo – Steven is an Entomologist & Vector Control Program Manager at the UCSF Malaria Elimination Initiative. Steven is based in Malawi and is interested in the control of vector-borne diseases. With nearly a decade of experience in both field and laboratory-based entomology, Steven has worked on malaria, arboviruses, and human African trypanosomiasis (sleeping sickness).

EM Studios – A game development studio with over 10 years industry experience based in Scotland. Since 2016, EM Studios have established a portfolio of educational games and software for African and Southeast Asian contexts in partnership with the Liverpool School of Tropical Medicine.

# Get in Touch

[insert UCSF contact details]
