# Supplementary material for: Evaluation of a digital entomological surveillance planning tool for malaria vector control: Three country mixed methods pilot study
Source: PLoS One. 2025 Mar 10;20(3):e0303915. doi: 10.1371/journal.pone.0303915 (PMC11892875; doi:10.1371/journal.pone.0303915)
Supplement: S2 Text — S2_example eSPT output. An example entomological surveillance planning document generated by the eSPT. (DOCX) [file pone.0303915.s002.docx]

| Entomological Surveillance Plan |  |
| --- | --- |
|  |  |
| 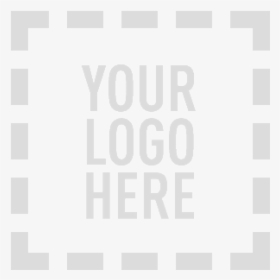09/04/2024  Organization/ authors name here | 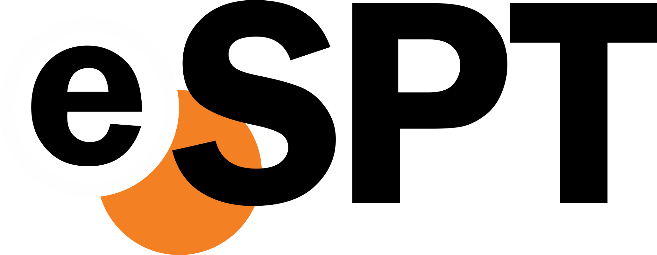 |

| 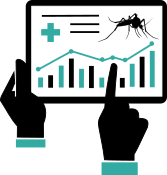  Should we deploy indoor residual spraying in Maputo district? | |
| --- | --- |
| Objectives | Minimum Essential Indicators |
| - In Maputo district between the 1st - 30th June 2022: 1. Measure vector occurrence and vector density to examine the presence of specific vector species and the relative vector composition, 2. Determine indoor resting density to examine the susceptibility of vectors to IRS based on their resting behaviors, and 3. determine the insecticide resistance status to examine the susceptibility of vectors to insecticide being considered for IRS. - Determine adult vector species composition - Determine adult vector behaviour - Determine insecticide resistance composition | Occurrence Density Indoor resting density Resistance frequency Resistance status Resistance intensity Resistance mechanism(s) (molecular or biochemical tests) |
| Sampling Methods | Analytical Techniques |
| Human landing catches (HLC) Indoor resting collections (IRC) Interception traps (window exit traps (WET)/barrier screen (BS)) Larval surveys (LS) | Molecular identification - PCR WHO tube assay Kdr PCR or biochemical assay |


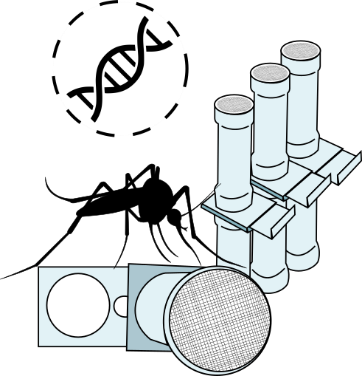


Authors notes

*Use this text box for additional explanatory text…*


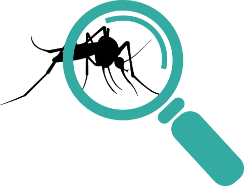
Sampling Design

| **Survey Type** | **Site Type** |
| --- | --- |
| Baseline survey | Focus site |

| **Sampling Site Description** |
| --- |
| Four villages in Maputo District each village will be considered a separate site (i.e., one village = one site). |

| **Sampling Method** | **Number of sampling units** | **Sampling frequency** | **Sampling unit allocation** |
| --- | --- | --- | --- |
| HLC Indoors and Outdoors | 4 structures (houses/outbuildings) in each site | 5 nights per month throughout the 5-month transmission season | Use a list of Village X structures and a random number generator to select a set number of sampling units (structures) |
| IRC, WETs and BS | 10 structures (houses/outbuildings) in each site | once per month throughout the 5-month transmission season | Use a list of Village X structures and a random number generator to select a set number of sampling units (structures). Select different structures each month to prevent residual PSC insecticide from affecting catches. |
| LS | All identified laval sites | 5 nights per month throughout the 5-month transmission season | Confirm (by morphological or molecular identification) that sampling reflects indoor resting vector populations (i.e., the same vector species). |

| **Estimated Cost of Plan** | **Budget** |
| --- | --- |
| $18,000.00 | $20,000.00 |


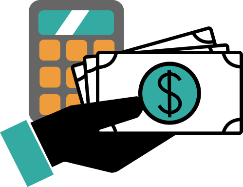


| **Budget line** | **Unit count** | **Unit cost** | **Total cost** |
| --- | --- | --- | --- |
| Human resources (collectors 2 per site) | 8 | $1,000.00 | $8,000.00 |
| Human resources (lab personal) | 4 | $2,000.00 | $8,000.00 |
| Travel to villiage sites | 1 | $2,000.00 | $2,000.00 |


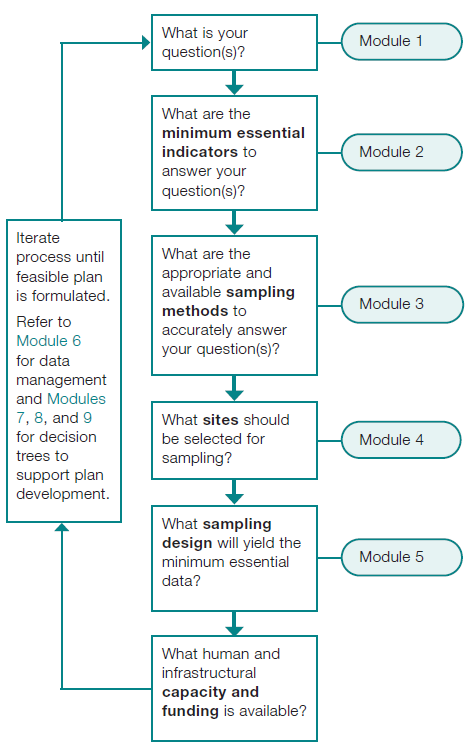
This plan has been developed using the Entomological Surveillance Planning Tool (ESPT)

The ESPT aligns with and aims to distill the World Health Organization (WHO) guidance into a decision-support tool for malaria programs to strengthen entomological surveillance and support cost-effective, locally tailored, evidence-based vector control. The ESPT also incorporates guidance from the President’s Malaria Initiative (PMI) and other technical partners and resources. The ESPT is a tool for planning entomological surveillance activities, interpreting and integrating entomological data with epidemiological data, and guiding programmatic vector control strategies. The ESPT includes practical approaches and minimum essential indicators to help answer programmatic questions about local transmission drivers, gaps in protection with current vector control interventions (e.g., insecticide resistance, outdoor biting, etc.), and how to select supplemental vector control interventions to address gaps. In turn, this data, in combination with epidemiological, intervention, and other data, will help malaria programs target and tailor vector control solutions, reduce vector populations and human-vector contact, and drive down transmission.

How does the eSPT work?

The eSPT supports the development of entomological surveillance plans by providing an interactive version of the ESPT framework. The eSPT is a unique and tailor-made word-processing software whereby the author(s) creates a tailored entomological surveillance plan (including minimum essential indicators and sampling design) based on a priority programmatic question. The eSPT automatically generates a record of decisions and inputs made by the author(s) as they work through the ESPT framework step by step; this record can be exported at any point as a formatted MS Word document. The MS Word document has been designed to support communication of the entomological surveillance plan to colleagues and partners.

*The eSPT does not make decisions for the author, the principle of the eSPT is to provide guidance on important considerations and give recommendations based on the objectives of the entomological surveillance.*

*Use the following template to help you expand the entomological surveillance plan to fit your communication needs…*

Background & Rationale

*Justification and rationale for conducting programmatic entomological surveillance. This could include:*

- *Recent trends in malaria cases or overview of malaria epidemiology*
- *Current and/or proposed types of vector control interventions*
- *Insecticide formulations and compounds used and/or proposed*
- *Description of malaria vector species present if known*
- *Description of malaria vector species insecticide resistance status if known*
- *Impact of vector control interventions on malaria transmission and disease outcomes if known*
- *Summary of knowledge gaps or outline the need for entomological surveillance*
- *List of partners/funders involved in entomological surveillance activities*

Indicators

*Outline the reasoning behind the selected indicators. Explain how these indicators will help answer the programmatic question and objectives. Identify which indicators are essential and which are supplementary.*

Sampling Methods & Analytical Techniques

*Provide further description of the sampling methods and analytical techniques selected e.g., standard operating procedures (SOPs). Outline the reasoning behind the selected sampling methods and analytical techniques.*

Sampling Site Selection

*Provide further description of survey type and site type. Outline the reasoning behind the sampling site design.*

Data Management Plan

*Outline the processes for collating and reporting data. This could include:*

- *Identify/ describe data collection forms*
- *Describe any adaptions to pre-existing data collection forms*
- *Provide data dictionaries for each form*
- *Outline procedures for data entry, cleaning, and quality assurance (e.g., standardized formatting, completeness and data verification)*
- *Outline procedures for data storage*
- *Outline any relevant data protection regulation and steps to ensure compliance*

Financial and Human Resources

*The financial and human resources required for successful implementation of the entomological surveillance plan. This could include:*

- *Current national and subnational human capacity devoted to entomological surveillance activities*
- *Overview of all partners active in entomological surveillance in support of the national malaria control programme*
- *Any current financial gaps or other resource gaps that may constrain implementation of the entomological surveillance plan*
- *Breakdown of the estimated cost of plan by budget lines e.g., travel, equipment, salary*
- *Possible cost saving activities*
- *Opportunities and strategies to secure co- funding*


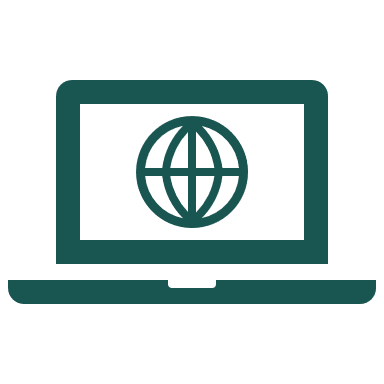
eSPT developed by:

[University of California San Francisco, Malaria Elimination Initiative](http://www.shrinkingthemalariamap.org)

[University of Notre Dame](https://biology.nd.edu/people/neil-f-lobo/)

[Liverpool School of Tropical Medicine](https://www.lstmed.ac.uk/about/people/dr-mike-coleman)

[EM Studios](https://www.emstudios.co.uk/)

Please contact us to learn more at [espt@ucsf.edu](mailto:espt@ucsf.edu)

[Download the ESPT](https://shrinkingthemalariamap.org/tool/entomological-surveillance-planning-tool-espt)
