# Supplementary material for: Evaluation of a digital entomological surveillance planning tool for malaria vector control: Three country mixed methods pilot study
Source: PLoS One. 2025 Mar 10;20(3):e0303915. doi: 10.1371/journal.pone.0303915 (PMC11892875; doi:10.1371/journal.pone.0303915)
Supplement: S3 Text — S3a_Malawi Knowledge & Self Efficacy assessment. Survey tool used in Malawi. (PDF) [file pone.0303915.s003.pdf]

Malawi eSPT Phase 2 Pilot: Knowledge & Self Efficacy Assessment

Please answer all questions to the best of your knowledge. Responses and scores will be anonymised. Individual scores will be kept private and will not be shared with your colleagues. Questions have been designed to evaluate the eSPT training session and relate to the learning objectives of the eSPT.

* Indicates required question

1. Today's Date *

Example: 7 January 2019

2. Participant ID - Please confirm your ID with one of the facilitators

3. Assessment stage *

Mark only one oval.

- Pre
- Post

4. What is the first step in designing an entomological surveillance plan? *

Mark only one oval.

- Identify the minimum essential indicators to collect
- Identify and formulate a specific programmatically relevant question
- Select your mosquito sampling methods
- Determine the number of sampling sites and sampling units

5. How would you best describe a minimum essential indicator? *

Mark only one oval.

- Any entomological surveillance indicator recommended by the WHO
- Any requisite indicator deemed indispensable to correctly measuring the outcome of interest and generating actionable data for decision-making
- Any requisite indicator deemed indispensable to understanding insecticide resistance among adult vectors
- Any entomological surveillance indicator that a program has the capacity and resources to collect

6. Why is it important to analyze and collect data from surveys on human behaviors together with entomological data? *

Mark only one oval.

- It allows for the identification of human drivers of exposure
- It allows for the identification of primary and secondary vectors in the area
- It helps to develop a better relationship with community members
- It gives the collectors an opportunity to get up and walk around

7. List 3 key considerations when selecting entomological sampling methods for a baseline survey. *

8. If using the human landing catch technique is not possible, which of the following sampling methods can be used as a proxy for data on vector occurrence, density, and biting behaviour? Select all that apply *

Tick all that apply.

- [ ] Human baited traps
- [ ] Indoor resting collections
- [ ] CDC light trap placed outside
- [ ] CDC light trap placed next to sleeping human
- [ ] CO2 modified CDC light trap
- [ ] Gravid traps
- [ ] Human odor baited traps

9. Which site type is important for measuring trends over time? *

Mark only one oval.

- ◯ Sentinel site
- ◯ Focus
- ◯ Target site

10. How would you best describe a gap in protection? *

Mark only one oval.

- ◯ Term used to describe open eaves in a house
- ◯ Term used to describe a human's potential exposure to malaria outside
- ◯ Term used to describe holes in an insecticide treated bed net
- ◯ Term used to describe a human's potential exposure to malaria due to a lack of effective malaria treatment and/or prevention being in place

11. A baseline survey to determine when and where people are exposed to vector biting has generated the following data on exposure risk (cumulative adjusted human biting rate). Give two recommendations for interventions based on this data. *

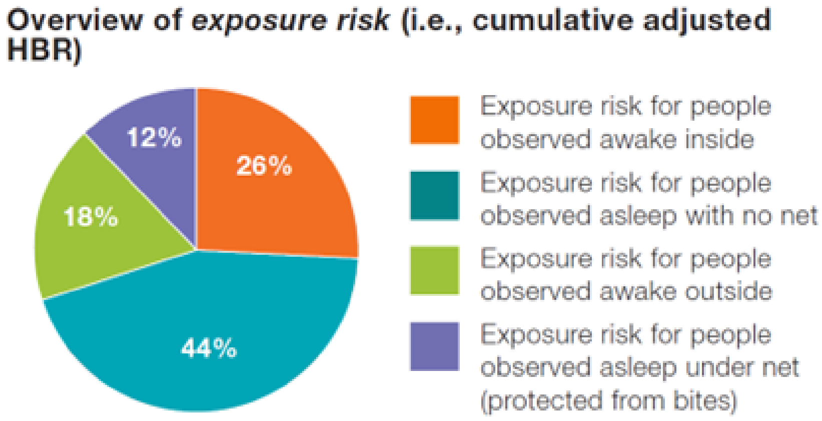

12. An entomological investigation has generated the following data on 3 vector species in area X. Species A was found in large numbers biting indoors and outdoors throughout the night. Species B was found in large numbers biting outside in the evening. Species C was found in smaller numbers biting outside in the evening. Species A & C larvae was found in small rain filled temporary bodies of water. Species B was found in more permanent bodies of water, including rice fields. The larvicide permitted for use in area X is known to be effective for 3 months. Based on this data and using a number to represent the month, which month would be the optimal time to deploy long lasting insecticide treated nets (LLINs)____ *

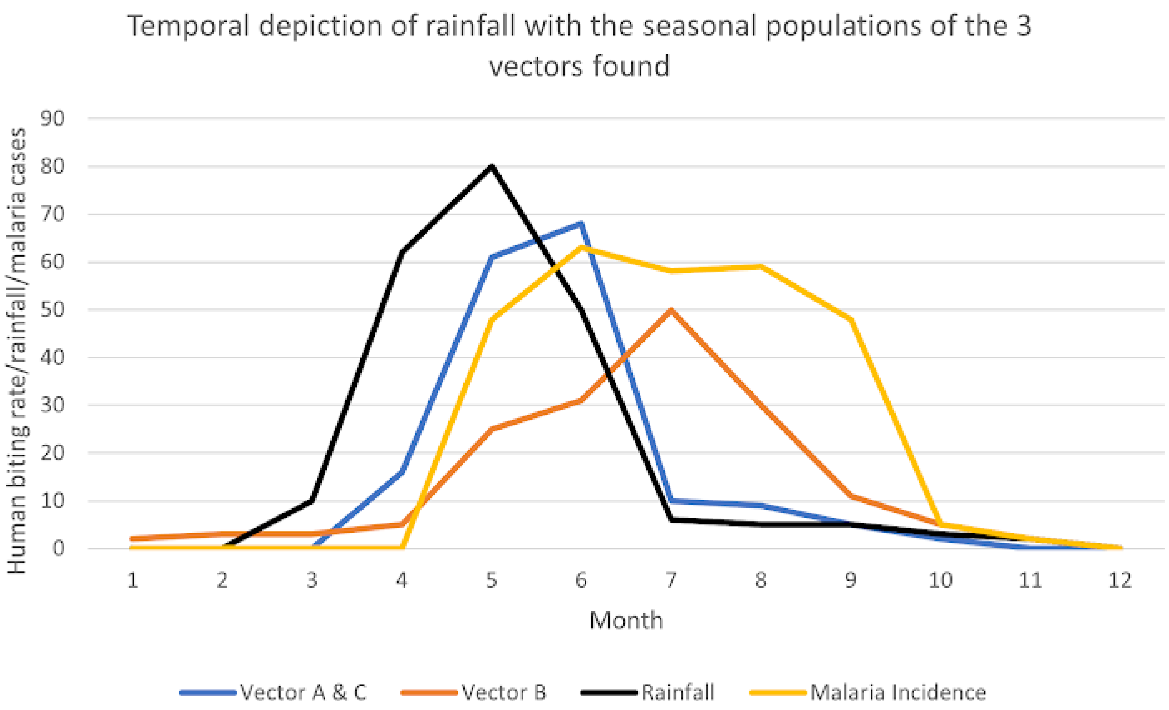

13. Based on the data from the previous question and using a number to represent the month, which two months would be the optimal time to deploy larvicide in rice fields____ & ____ *

Case Study Questions

Malaria is not changing in Mangochi district in Malawi. Data suggests good coverage and usage of LLINs above 85%. The NMCP would like to understand why malaria is not going down and would like to know the present efficacy of LLINs, the gaps in protection (that result in continued malaria) as well as if IRS will work.
The budget for this entomological investigation is $50,000 over 1 year.

14. What are the minimum essential indicators to determine whether IRS should be introduced in Mangochi district? Select all that apply *

Tick all that apply.

- [ ] Vector occurrence
- [ ] Vector density
- [ ] Seasonality (adult vectors)
- [ ] Larval habitat availability
- [ ] Larval habitat occupancy
- [ ] Human biting rate
- [ ] Biting time
- [ ] Biting location
- [ ] Indoor resting density
- [ ] Resistance frequency
- [ ] Resistance status

15. Which of the following are advisable sampling method(s) to determine whether IRS should be used? Select one answer *

Mark only one oval.

- ( ) Indoor resting collections and larval surveys
- ( ) Human landing catch or proxy
- ( ) Indoor resting collections and human landing catch or proxy
- ( ) Indoor resting collections, human landing catch or proxy and larval surveys

16. Why did you choose your answer to the previous question? *

17. True or false, outdoor human landing catches would not provide useful data for the Mangochi district case study? *

Mark only one oval.

- True
- False

18. Which of the following analytical technique can be used to determine the mechanism of resistance? *

Mark only one oval.

- PCR
- Cone bioassays
- WHO tube bioassays
- CDC bottle assay

19. Which of the following objectives relates to the identification of gaps in protection caused by human behaviour? Select all that apply. *

Tick all that apply.

- [ ] Determine adult vector species composition
- [ ] Determine adult vector species seasonality/ appropriate timing of interventions
- [ ] Determine impact of adult vector control interventions (e.g., IRS, LLINs)
- [ ] Determine adult vector behaviour
- [ ] Determine insecticide resistance composition
- [ ] Determine malaria transmission potential
- [ ] Determine host preference of adult vectors
- [ ] Determine larval composition
- [ ] Determine access to, quality, and/or usage of interventions
- [ ] Determine human behaviours and associated risk factors

Occupational Self-efficacy and Confidence Assessment

Please select the number that best reflects how you honestly feel about each statement below. Only select one number for each statement.

20. I can remain calm when facing difficulties in entomological surveillance planning because I can rely on my abilities. *

Mark only one oval.

	1	2	3	4	5	6	
Not	◯	◯	◯	◯	◯	◯	Completely true

21. When I am confronted with a problem in entomological surveillance planning, I can usually find several solutions. *

Mark only one oval.

	1	2	3	4	5	6	
Not	◯	◯	◯	◯	◯	◯	Completely true

22. Whatever comes my way in entomological surveillance planning, I can usually handle it. *

Mark only one oval.

	1	2	3	4	5	6	
Not	◯	◯	◯	◯	◯	◯	Completely true

23. My past experiences in entomological surveillance planning have prepared me well for my occupational future. *

Mark only one oval.

	1	2	3	4	5	6	
Not	◯	◯	◯	◯	◯	◯	Completely true

24. I meet the goals that I set for myself in entomological surveillance planning. *

Mark only one oval.

	1	2	3	4	5	6	
Not	◯	◯	◯	◯	◯	◯	Completely true

25. I feel prepared for most of the demands in entomological surveillance planning. *

Mark only one oval.

	1	2	3	4	5	6	
Not	◯	◯	◯	◯	◯	◯	Completely true

26. I feel confident in my ability to design an entomological surveillance plan. *

Mark only one oval.

	1	2	3	4	5	6	
Not	◯	◯	◯	◯	◯	◯	Completely true

27. I feel confident in my ability to identify and formulate a specific programmatically relevant question for entomological surveillance. *

Mark only one oval.

	1	2	3	4	5	6	
Not	◯	◯	◯	◯	◯	◯	Completely true

28. I feel confident in my ability to select appropriate minimum essential indicators based on my entomological surveillance question. *

Mark only one oval.

	1	2	3	4	5	6	
Not	◯	◯	◯	◯	◯	◯	Completely true

29. I feel confident in my ability to select appropriate sampling methods/ entomological techniques based on my entomological surveillance question. *

Mark only one oval.

	1	2	3	4	5	6	
Not	◯	◯	◯	◯	◯	◯	Completely true

30. I feel confident in my ability to develop an appropriate sampling design based on my entomological surveillance question. *

Mark only one oval.

	1	2	3	4	5	6	
Not	◯	◯	◯	◯	◯	◯	Completely true

31. I feel confident in my ability to develop an affordable entomological surveillance plan. *

Mark only one oval.

	1	2	3	4	5	6	
Not	◯	◯	◯	◯	◯	◯	Completely true

32. I feel confident in my ability to communicate my entomological surveillance plan to stakeholders/ decision makers. *

Mark only one oval.

	1	2	3	4	5	6	
Not	◯	◯	◯	◯	◯	◯	Completely true

This content is neither created nor endorsed by Google.

Google Forms
