# Supplementary material for: Evaluation of a digital entomological surveillance planning tool for malaria vector control: Three country mixed methods pilot study
Source: PLoS One. 2025 Mar 10;20(3):e0303915. doi: 10.1371/journal.pone.0303915 (PMC11892875; doi:10.1371/journal.pone.0303915)
Supplement: S6 Text — S4_FGD Topic Guide_eSPT ICT Pilot Study. Focus group discussion topic guide. (DOCX) [file pone.0303915.s006.docx]

# Phase 2 ESPT ICT Pilot Study

**Focus group discussion topic guide**

**FGD Set Up**

- Ensure all participants are in frame for the video recording (Ethiopia & Mozambique only).
- Place audio recording devices in a central position so they are likely to pick up everyone’s voice.
- Aim for a maximum of 8 participants in the FGD. Aim to group participants based on relationship dynamics and job roles to encourage them to speak freely. Record the reasoning behind groupings.

**Opening Discussion**

***What is the planning process for entomological surveillance?***

Tell us about how entomological surveillance planning is conducted in your organisation? - (Probe on what planning process looks like, guidelines/frameworks used, based on programmatic questions Y/N and why, who formulates the questions, who determines the type of activities and/or methods used, challenges)

**Perceived Ease of Use + Determinants**

***What software is currently used in the planning process for entomological surveillance?***

Do you use any software or digital tools for entomological surveillance planning? (ensure discussion stays focused on planning activities, we’re not asking for software used for data collection/ management)

Tell us about the software/digital tools you use for entomological surveillance (ES) planning? - (probe on limitations and benefits of software used to develop/communicate ES plans, be clear you are not asking for software used for data collection/ management)

How much control do you have over the software you use for ES planning? - (probe on who influences their choice/use of software)

***How usable is the ESPT ICT?***

Overall, what did you think of the ESPT ICT today? (probe on ease of use, effort required to learn how to use the tool, overall likes and dislikes)

How did you feel using the ESPT ICT today? Do you feel you have the resources needed to use the ESPT ICT? - (probe on training, hardware requirements, confidence, anything missing)

**Perceived Usefulness + Determinants**

***How useful is the ESPT ICT?***

Thinking about the 2-day training you have attended, how useful was the ESPT ICT during the training?

Thinking about your role in entomological surveillance, how useful is the ESPT ICT to you? - (probe on productivity, job effectiveness, communication, need/demand for tool, practical uses, what is it about the software that is useful)

What did you think of the question-based approach to entomological surveillance planning? Were you familiar with this approach before today? (probe on practical use in job, familiarity with approach, perceived ease or difficulty in applying, apprehensions/anticipated challenges)

Thinking about the people you work with who are not here today, what would they think about the ESPT ICT?

**Behavioural Intention**

(Ensure participant clarify whether their behavioural intention relates to the ESPT document, the ESPT ICT, the ESPT facilitated training, or any of these combined.)

***Do you plan to use the ESPT ICT in the future?***

Do you plan to use the ESPT again as part of your professional role? How would you use the ESPT? - (probe on if/how they would specifically use the ESPT ICT, how they would use the word document output)

What would need to happen for you to use the ESPT in your professional role? – (probe on changes to the ESPT ICT, promotion, training required, technical support)

What impact, if any, do you think that the ESPT ICT could have on entomological surveillance design and planning in your organisation? - (probe on facilitators/inhibitors of impact)

Do you have any other feedback for us on the ESPT ICT?
