# Supplementary material for: Evaluation of a digital entomological surveillance planning tool for malaria vector control: Three country mixed methods pilot study
Source: PLoS One. 2025 Mar 10;20(3):e0303915. doi: 10.1371/journal.pone.0303915 (PMC11892875; doi:10.1371/journal.pone.0303915)
Supplement: S7 Text — S5_IDI Topic Guide_eSPT ICT Pilot Study. In-depth interview topic guide. (DOCX) [file pone.0303915.s007.docx]

# Phase 2 ESPT ICT Pilot Study

**Follow up interview topic guide**

**Opening question**

Tell me about what you’ve been doing professionally/ workwise since you attended the ESPT training course? (Probe – have you done anything specifically around entomological surveillance, why or why not)

**Perceived Changes in Knowledge & Work Practices**

Has your knowledge of entomological surveillance changed in any way since you participated in the course? (Probe – if yes what do you think triggered the change)

Has your approach to entomological surveillance changed in any way since you participated in the training? (Probe – if yes what do you think triggered the change)

What tools do you use for entomological surveillance? (Probe – software, guidelines)

Have the tools (e.g. software, guidelines) you use for entomological surveillance changed since you participated in the training? (Probe – if yes what do you think triggered the change)

**ESPT Decision Support Tools Perception and Use**

Have you used the ESPT document in your professional role since participating in the training course? (Probe – how did you use the tool, who did you use it with, what benefit did that have if any)

Have you used the ESPT ICT in your professional role since participating in the training course? (Probe – how did you use the tool, who did you use it with, what benefit did that have if any)

Do you plan to use any of the ESPT decision support tools, such as the ESPT document or ESPT ICT or a combination of both in the future? (Probe – how will you use the tool, who will you use it with, what benefit do you think it will have if any)

If you have not used and/or do not plan to use any of the ESPT decision support tools in the future, can you please explain why that is?

Do you feel you would be supported by your superiors/ institute/ funders in using any of the ESPT decision support tools?

Would you feel confident in using any of the ESPT decision support tools outside a facilitated training session?

Do you feel motivated to use any of the ESPT decision support tools in the future? (Probe – can you explain why you answered yes, no)

Is there anything you would like to tell the developers of the ESPT ICT? (Probe – any changes/ additions to the software you would like to see implemented?)

**Participant Details**

How old are you? If you don’t want to give your exact age you can give an age range.

What is your job title?

What are your primary responsibilities in your job?

What education/ training and you undertaken for your current job?

How confident do you feel using new software/ technology in your professional role?
