# Supplementary material for: Evaluation of a digital entomological surveillance planning tool for malaria vector control: Three country mixed methods pilot study
Source: PLoS One. 2025 Mar 10;20(3):e0303915. doi: 10.1371/journal.pone.0303915 (PMC11892875; doi:10.1371/journal.pone.0303915)
Supplement: Attachment — Submitted filename: eSPT Manuscript_response to reviewers_PlosOne_Dec2024.docx [file pone.0303915.s011.docx]

Dear Dr Muzafar Riyaz,

Thank you for the opportunity to submit a revised version of our manuscript titled ‘Evaluation of a Digital Entomological Surveillance Planning Tool for Malaria Vector Control: Three Country Mixed Methods Pilot Study.’ Please find within our numbered response to points raised by the reviewers.

References to lines in the manuscript relate to the simple mark-up view.

**Reviewer #4:**

With respect, this research was re-examined in terms of statistics and content.
Please make the following corrections:

**4.1** The study employs an uncontrolled design, meaning there is no control group to compare the impact of the eSPT workshops. This makes it difficult to determine if the observed knowledge gains and changes in self-efficacy were due to the intervention or other external factors.

**Response:** The authors acknowledge this limitation. Additional text has been added to methods under study type (line 133-137) to clarify the goals of the pilot study.

**4.2** While the authors acknowledge the small sample size, it raises concerns about the generalizability of the findings.

**Response:** Rephrased the conclusion to state ‘Interactive digital toolkits **could** be an engaging, efficient, and accessible way to build research and surveillance capacity…’ to make it clearer that the findings are not generalizable, and that further research is recommended.

**4.3** Additionally, the study only includes highly educated participants with strong prior knowledge, which may not reflect the challenges faced by less experienced or less-educated users.

**Response:** Text added to limitations line 646-651 recommended further evaluation of the eSPT among participants with less experience in entomological surveillance planning.

**4.4** The follow-up period of only one month may be insufficient to assess the long-term impact of the eSPT on work practices and knowledge retention. Longer-term studies could provide more insights into whether participants continue using the tool and how it influences their work over time.

**Response:** This is acknowledged in the limitations line 635

**4.5** The workshops did not align with the participants' entomological surveillance planning cycles, which could have negatively influenced their perceptions of the tool’s relevance. This lack of alignment with real-world activities reduces the study's ecological validity.

**Response:** This is acknowledged in the limitations line 632-634

**4.6** The eSPT is designed specifically for malaria vector control, limiting its usefulness to researchers or programs dealing with other vector-borne diseases. Some participants indicated that broader applicability would enhance the tool’s value.

**Response:** Inclusion of other vector borne diseases was not feasible within the eSPT development budget and project timeframes. Given the findings of the study, this content expansion will be prioritised if additional funding is obtained for further software development. Funding to further develop the eSPT is being pursued by study authors.

**4.7** Despite the qualitative nature of much of the data, there are attempts to generalize the findings beyond the study sample. The authors caution against generalizing but then draw conclusions that extend beyond the scope of the study.

**Response:** Rephrased the conclusion to state ‘Interactive digital toolkits **could** be an engaging, efficient, and accessible way to build research and surveillance capacity…’

**4.8** The involvement of local researchers in data collection was intended to reduce cultural and language biases, but there remains a potential bias due to the participants' familiarity with these researchers, which may influence their responses.

**Response:** text added to limitations line 656-659 acknowledging the potential response bias.

**4.9** Despite participants' expressed enthusiasm for the tool, the actual reported use of the eSPT was minimal in the month following the workshop. This raises questions about the practical utility of the tool outside the controlled environment of the training sessions.

**Response:** further discussion added line 623-628, reflecting on findings from the FGDs and interviews.

**4.10** Depending on the nature of the study, you can benefit from and cite the following articles:
- Knockdown resistance (kdr) associated organochlorine resistance in mosquito-borne diseases (Culex quinquefasciatus): Systematic study of reviews and meta-analysis

- Knockdown resistance (kdr) Associated organochlorine Resistance inmosquito-borne diseases (Anopheles culicifacies): Systematic reviews study

- Knockdown resistance (kdr) Associated organochlorine Resistance in mosquito-borne diseases (Culex pipiens): Systematic study of reviews and meta-analysis

- Knockdown Resistance (kdr) Associated Organochlorine Resistance in Mosquito-Borne Diseases (Anopheles subpictus): Systematic Review Study

**Response:** thank you for the recommendation

The author would like to thank the reviewer for their expertise and time.

**Reviewer #5:**

Abstract
**5.1** The phrase “contributed to shrinking the malaria map” is vague and colloquial for an academic abstract. Use specific language, such as “has led to significant reductions in malaria incidence across endemic regions.”

**Response:** edit made to abstract line 31-32

**5.2** The sentence “an Entomological Surveillance Planning Tool (ESPT) was developed to distil normative guidance into an operational decision-support tool...” blends past and present tenses ambiguously. To keep focus and clarity, revise as “The ESPT was designed to translate normative guidance into an operational tool that supports...”

**Response:** edit made to abstract line 34-35

**5.3** The phrase “users found the software easy and enjoyable to navigate” is subjective and lacks rigorous scientific measurement. It would be scientifically sound to rephrase this based on the specific positive feedback mechanisms observed, e.g., “Users reported high usability scores and satisfaction with the interface.”

**Response:** edit made to abstract line 46-47

**5.4** “A mixed-methods, uncontrolled, before and after study...” is somewhat confusing without further clarification of each method used and their alignment with study goals. A more detailed description, like “A mixed-methods design was employed, combining pre- and post-intervention surveys with in-depth interviews to assess...”,

**Response:** edit made to abstract line 40-41

**5.5** Objectives are sometimes introduced ambiguously. For instance, “to support roll-out of the ESPT’s question-based entomological surveillance planning an interactive digital toolkit, eSPT, was developed.” This lacks clear flow. Streamline objectives by specifying roles and tools concisely: “To facilitate ESPT implementation, an interactive digital toolkit (eSPT) was created to support question-based surveillance planning.”

**Response:** edit made to abstract line 35-37

Introduction
**5.6** The phrase “shrinking the malaria map” is colloquial and too informal for a scientific introduction.

**Response:** edit made to introduction line 62-63

**5.7** The description of ESPT development is long-winded and could be more concise, especially in a background section.

**Response:** edit made to introduction line 74-76

**5.8** Sentences like “An Entomological Surveillance Planning Tool (ESPT), developed in 2018 by the Malaria Elimination Initiative (MEI) at the University of California, San Francisco (UCSF) and the University of Notre Dame (UND), with guidance from a technical working group, distills normative guidance...” are complex and contain multiple clauses. Break down long sentences to avoid overwhelming readers.

**Response:** edit made to introduction line 74-76

**5.9** Details about the user-centered design process and usability testing are long and distract from the primary study aim. Summarise this process in one sentence.

**Response:** Details of the user-centered agile software development process are important as user-centred design methods are often ill-defined and varied. As the discussion includes literature on the association between user-centered agile software development approaches and product usability, the authors feel it is important to provide readers with clear definition of the eSPT’s user-centered development approach, including who was involved in the user testing and the methods used to capture user experience and feedback.

**5.10** While the introduction provides background, it lacks a strong statement about the study's significance and how it contributes to the current body of knowledge. Conclude the introduction with a clear statement of the study’s relevance, such as: “This evaluation seeks to understand the eSPT’s impact on knowledge acquisition and usability, informing future adoption of digital planning tools in vector control.”

**Response:** edit made to introduction line 105-108

**5.11** Phrases like “contextually-relevant” and “question-based entomological surveillance planning” lack clear definitions. Replace with precise language or provide definitions.

**Response:** edit made to introduction line 65-66 – definition added for contextually-relevant. “Question-based” has been deleted as this is covered under the broader concept of evidence-based vector control.

Methods
**5.12** The terms “countries,” “sites,” and “locations” are used inconsistently, making it difficult to follow the study structure.

**Response:** terms replaced with “countries/country” throughout for consistency

**5.13** The study design, a “mixed-methods, uncontrolled, before and after study,” is briefly mentioned without explaining its relevance or rationale for this approach.

**Response:** paragraph added to methods line 133-137

**5.14** It is unclear why certain data, such as the knowledge retention questionnaires, were collected “at least 30 days following the workshop.” Specify the rationale for the 30-day interval.

**Response:** rationale added to methods line 226-230

**5.15** Participant recruitment is described broadly; however, criteria specifics (e.g., what defines a “target user”) are unclear. Refine criteria to clarify who qualified as a “target user.”

**Response:** edit made to method line 144-145 to clarify that ‘target user’ is used to refer to study participants

**5.16** The use of statistical tests, such as “dependent samples paired t-test or its non-parametric equivalent, Wilcoxon signed-rank test,” could be clearer. Provide context on when each test was applied, such as, “A dependent samples paired t-test was used to analyse normally distributed data; otherwise, the Wilcoxon signed-rank test was applied.”

**Response:** edits made to data analysis lines 244-248 to provide context for each test.

“A dependent samples paired t-test was employed to analyse knowledge acquisition data, which exhibited normal distribution. In contrast, self-efficacy data, which deviated from normality, were analysed using the Wilcoxon signed-rank test.”

Results
**5.17** Results Interpretation: Discuss each finding (e.g., “slightly significant” can be replaced with a more specific interpretation) and consider mentioning potential contributing factors for non-significant findings.

**Response:** Edits made to lines 271-273 to replace "slightly significant" with "significant." Further, potential contributing factors for non-significant findings have been explored in the discussion section.

**5.18** Use terms like “significantly increased” or “no significant difference observed” instead of “slightly significant.” Clearly state whether a statistical test was parametric or non-parametric.

**Response:** Edits made to lines 271-273 to replace "slightly significant" with "significant." Additionally, lines 244-248 were revised to clarify that parametric tests analysed normally distributed knowledge acquisition data, while the Wilcoxon signed-rank test analysed non-normally distributed self-efficacy data.

**5.19** Use of “Overall” and “Country Level” Comparison: Consistently differentiate between overall and country-specific results by specifying these terms in each relevant sentence. For instance, “No significant difference was observed in knowledge in Malawi (p = 0.41), while overall results showed significant retention.”

**Response:** Edits made to lines 288-289 to show distinction between overall and country-specific results.

**5.20** Introduce each quote by paraphrasing or summarizing the point before citing verbatim, making the qualitative data more digestible.

**Response:** quotes are used illustratively; summaries of the quotes can be found in the narrative interpretation, as is the standard practice for presenting qualitative research. The authors believe that further additions of paraphrased quotes would not improve readability.

**5.21** Indicate the number of participants for FGDs and interviews at the beginning of each objective’s section to set the context.

**Response:** we are not sure what the reviewer is referring to by ‘objective section’ – the number of FGD and interview participants per county is clarified at the start of the results section titled ‘Objective 3: Technology Acceptability’ to provide context to the findings.

**5.22** Conclude each objective’s section with participant recommendations for software improvements, organizational support, or resource needs.

**Response:** we are not sure what the reviewer is referring to by ‘objective section’ – we are also unsure of the rationale for repeating participants recommendations at the end of the results section or at the end of each theme.

Discussion
**5.23** Statements like "The positive response to the eSPT as a learning and teaching tool can be explained by the positive affective-motivational states..." need more empirical backing or citations beyond the general assertion. Rephrase or add references to justify the claim.

**Response:** reference 19 and 20 are cited in the proceeding sentence to justify the claim quoted in the reviewers comment.

**5.24** The statement "The modest increase in knowledge suggests that the eSPT contained new information..." is vague. Specify the metrics of the "modest increase" to clarify the level of knowledge change and its potential causes.

**Response:** edit made to discussion line 566-567

**5.25** Phrases like "could be a useful tool" and "the eSPT may therefore support..." use conditional phrasing, which is appropriate, but statements regarding the eSPT’s impact on capacity-building or filling gaps in expertise are too generalized.

**Response:** rephrased the conclusion to state ‘Interactive digital toolkits **could** be an engaging, efficient, and accessible way to build research and surveillance capacity…’

**5.26** The statement about limited use of the eSPT post-workshop is significant but lacks further analysis. Consider exploring reasons beyond niche focus, such as environmental or organizational barriers. Discuss potential reasons for low engagement beyond niche focus, like accessibility issues, technical challenges, or competing priorities.

**Response:** further discussion added line 623-628, reflecting on findings from the FGDs and interviews.

We’d like to thank the reviewer for their helpful comments, especially suggestions for rephasing the text. We very much appreciate their time and expertise and hope we have adequately addressed the comments raised.
