# Supplementary material for: Evaluation of a digital entomological surveillance planning tool for malaria vector control: Three country mixed methods pilot study
Source: PLoS One. 2025 Mar 10;20(3):e0303915. doi: 10.1371/journal.pone.0303915 (PMC11892875; doi:10.1371/journal.pone.0303915)
Supplement: Attachment — Submitted filename: eSPT Manuscript_response to reviewers_PlosOne.docx [file pone.0303915.s010.docx]

Dear Dr Delfina Fernandes Hlashwayo,

Thank you for the opportunity to submit a revised version of our manuscript titled ‘Evaluation of a Digital Entomological Surveillance Planning Tool for Malaria Vector Control: Three Country Mixed Methods Pilot Study.’ Please find within our numbered response to points raised by the academic editor and reviewers.

Editor’s comments:

1. Manuscript title page, list of authors, and supporting information files names and citations have been updated in line with journal style requirement.
2. PLOS’ questionnaire on inclusivity in global research has been completed and included in the submission.
3. Statement clarifying the funders had no role in the study added to the financial disclosure.
4. ‘eSPT development team’ is not a commercial company. Authors CH, SG, MC, and NL are all employed by an academic institute as stated under author affiliations. Statement was included under competing interests to ensure transparency over the authors contributions to the design and distribution of the eSPT and to clarify that these authors do not stand to financially gain from uptake and use of the eSPT given its status as a free to use software. Text under competing interest has been updated to avoid confusion.

### Competing interests

CH, SG, MC, NL were involved in the design and distribution of the eSPT, as the tool is free to use and distribute, they do not stand to gain financially from the publication of this manuscript.

5. Anonymised responses to the knowledge and self-efficacy assessment have been included as supplementary material (S6). We can not upload focus group discussion and interview transcripts to a public repository or as supplementary material as this would breach compliance with the protocol approved by the research ethics board. The availability of data and materials statement in the manuscript has been edited as follows:

### Availability of data and materials

Anonymized response data from the knowledge and self-efficacy questionnaire is provided in the supplementary material (S6). Transcripts generated and/or analyzed during the current study are available from the corresponding author on reasonable request. Assessment instruments and topics guides are included as additional files.

Reviewer 2 comments:

1. *In lines 329 to 360, there are identifiable format changes needed, are plans in place to address?*

Inclusion of customisable document formats was not feasible within the eSPT development budget and project timeframes. Given the findings of the study, this functionality will be prioritised if additional funding is obtained for further software development. Funding to further develop the eSPT is being pursued by study authors.

2. *Can cost savings/benefits be estimated by planning efficiency and time resources utilizing the tool in planning?*

Economic evaluation was not within scope of the study, any inclusion of cost saving/benefits resulting from the eSPT would be speculative.

3. *What obstacles are there for acceptance/utilization of the tool by country MOH/NMCP and plans to address.*

The primary obstacle for acceptance/utilization of the eSPT by government organisations reported by study participants was poor IT infrastructure at district/ provincial level to support devolution of planning activities. This is articulated in the results section.

Reviewer 3 comments:

1. We acknowledge the small sample size of the study, however, note that the sample size was adequate for the statistical methods used and was representative given the small population of people in each country that met the study inclusion criteria.
2. We’d like to thank reviewer 3 for their insightful comments. The authors agree that further development of the software tool should take a more participatory approach with target end users, including target end users at different decision-making levels, enabling the development of more accessible and useful functionality.

We’d like to thank the reviewers for their time and expertise and hope we have adequately addressed the comments raised.
